# Electroporation-Based Treatments in Small Animal Veterinary Oral and Maxillofacial Oncology

**DOI:** 10.3389/fvets.2020.575911

**Published:** 2020-09-29

**Authors:** Ana Nemec, Nina Milevoj, Urša Lampreht Tratar, Gregor Serša, Maja Čemažar, Nataša Tozon

**Affiliations:** ^1^Small Animal Clinic, Veterinary Faculty, University of Ljubljana, Ljubljana, Slovenia; ^2^Institute of Oncology Ljubljana, Ljubljana, Slovenia

**Keywords:** electroporation, electrochemotherapy (ECT), gene electrotransfer, oral tumors, dogs, cats

## Abstract

Electroporation is a method of inducing an increase in permeability of the cell membrane through the application of an electric field and can be used as a delivery method for introducing molecules of interest (e.g., chemotherapeutics or plasmid DNA) into cells. Electroporation-based treatments (i.e., electrochemotherapy, gene electrotransfer, and their combinations) have been shown to be safe and effective in veterinary oncology, but they are currently mostly recommended for the treatment of those solid tumors for which clients have declined surgery and/or radiotherapy. Published data show that electroporation-based treatments are also safe, simple, fast and cost-effective treatment alternatives for selected oral and maxillofacial tumors, especially small squamous cell carcinoma and malignant melanoma tumors not involving the bone in dogs. In these patients, a good local response to treatment is expected to result in increased survival time with good quality of life. Despite emerging evidence of the clinical efficacy of electroporation-based treatments for oral and maxillofacial tumors, further investigation is needed to optimize treatment protocols, improve clinical data reporting and better understand the mechanisms of patients' response to the treatment.

## Basic Principles of Electroporation-Based Treatments and General Applications in Oncology

### Electroporation

Electroporation or electropermeabilization describes an increase in the permeability of the cell membrane due to the application of an electric field. The delivery of short high-voltage electrical pulses causes the formation of permeable structures in the cell membranes, thus allowing the passage of water-soluble ions and molecules into the cytosol (1). The key factor for successful permeabilization is the induced transmembrane voltage, which is generated in the presence of an external electrical field due to the difference in the electrical properties of the membrane and the external medium (2). The increase in cell membrane permeability may be transient (reversible electroporation) or may directly lead to cell death (irreversible electroporation), depending on the time of exposure of the cells to electrical pulses and the strength of the electric field. Cell death in irreversible electroporation may result from permanent disruption lysis of the cell membrane or the destruction of cellular homeostasis (3). Irreversible electroporation can be used in medicine as a sole treatment, and reversible electroporation can be combined as a delivery method for uploading molecules of interest (e.g., chemotherapeutics or plasmid DNA) into the cells (4, 5).

**Figure 1:**
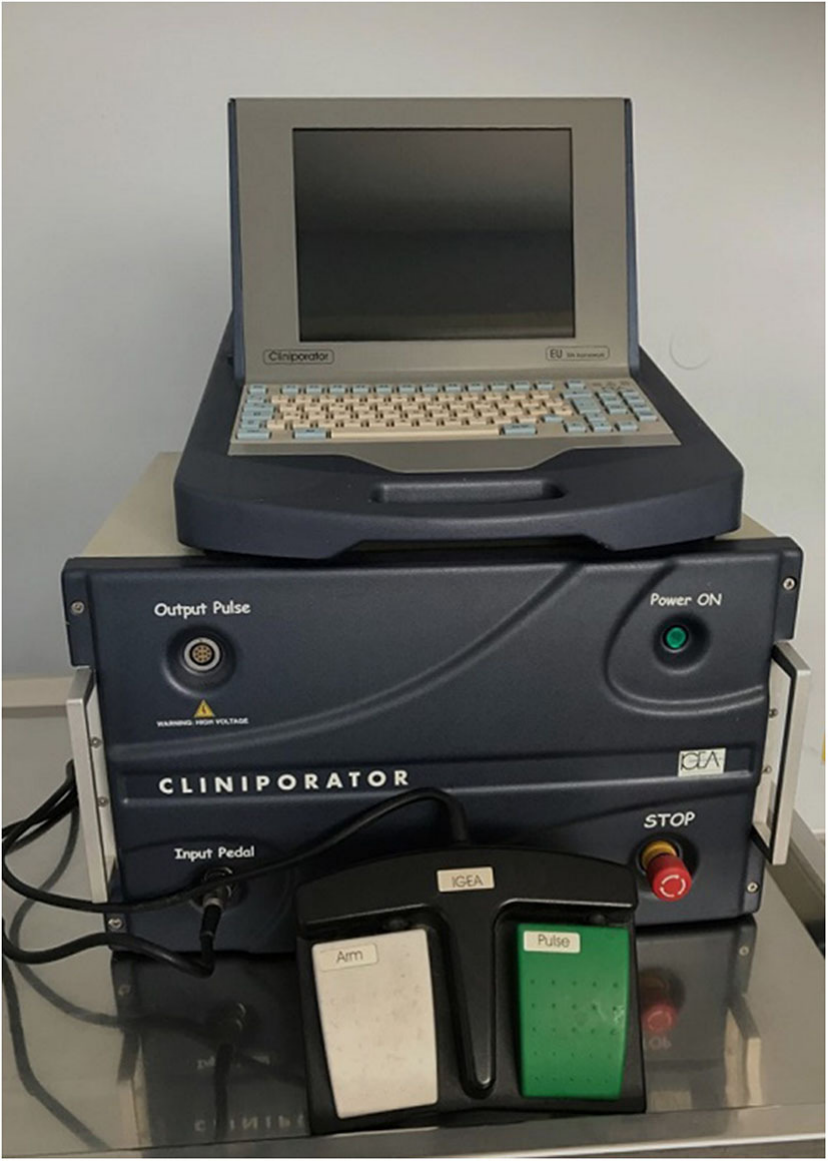
Generator of electrical pulses (Cliniporator, Igea s.r.l., Carpi, Italy).

**Figure 2:**
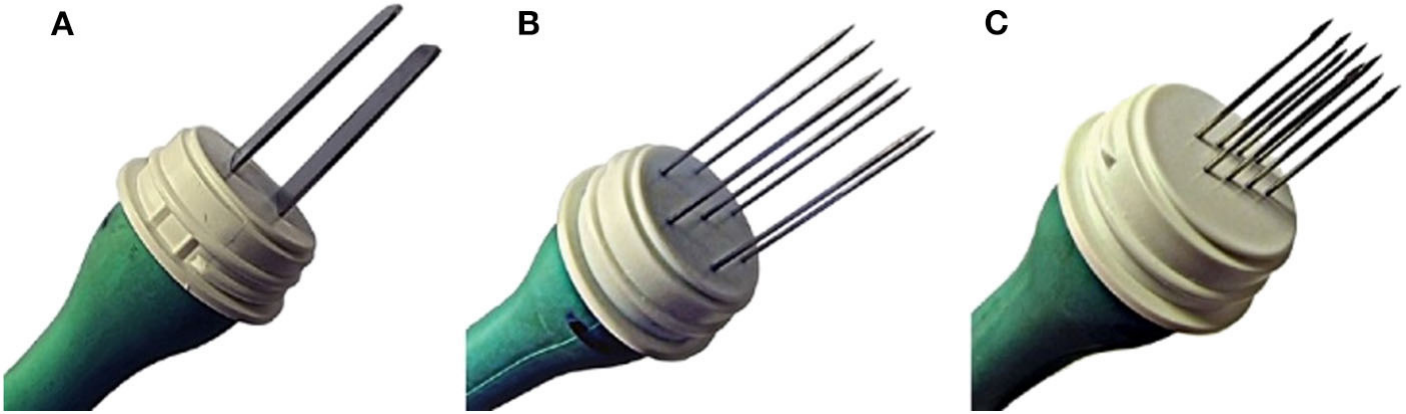
Standard types of electrodes, used for electroporation. **(A)** Non-penetrating plate electrodes, **(B)** penetrating hexagonal electrodes, **(C)** penetrating needle row electrodes (all from Igea s.r.l., Carpi, Italy).

**Figure 3:**
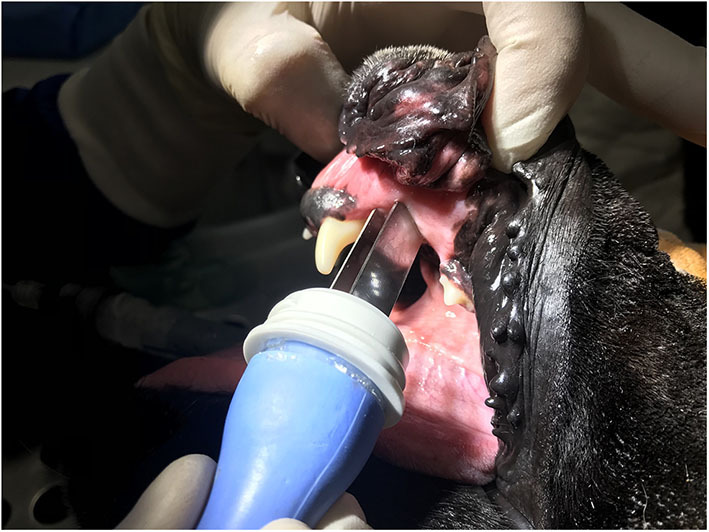
The treatment of a canine oral squamous cell carcinoma with electrochemotherapy with intravenous application of bleomycin, followed by electrical pulse delivery with plate electrodes.

Several pulse generators (Figure 1) with different types of electrodes (Figure 2) are currently available on the market. When reversible electroporation is used for drug and gene delivery, electrical parameters must be adjusted for the delivery of the desired molecules into target tissues (1). Efficient cell membrane electroporation depends on establishing a sufficiently high electric field in the target tissue. In general, short high-voltage pulses are applied for the insertion of smaller molecules (e.g., chemotherapeutics), and larger molecules require pulses of longer duration that are either low-voltage or a combination of high- and low-voltage (6). There are two standard types of electrodes used for electroporation: penetrating (e.g., needle row and hexagonal) and non-penetrating (e.g., plate) (7) (Figure 2). The electrodes are selected individually, depending on the depth of the tumor nodule and the properties of the target tissue. In general, plate electrodes are used for superficial tumors (Figure 3), and needle or hexagonal electrodes are used for deeper tumors to achieve electroporation throughout the entirety of the tumor (8). In recent years, with the advancement of electroporation-based treatments, new types of electrodes are being produced (e.g., the single-needle electrode), which enables variable geometry specialized for electroporation of deep-seated tumors (9), and a multi-electrode array with pins for gene delivery to the skin (10).

### Electrochemotherapy

Electrochemotherapy (ECT) is a local ablative method for the treatment of solid tumors that combines reversible electroporation and chemotherapy. Hydrophilic chemotherapeutics (bleomycin or cisplatin) can be administered either intravenously or intratumorally, and electric pulses are delivered directly to the tumor. Cells in the tumors become more permeable to chemotherapeutic agents that would otherwise have difficulty entering the cells (11, 12). This results in high concentrations of the intracellular chemotherapeutic agents and, consequently, up to 70 times (cisplatin) or thousands of times (bleomycin) more potent antitumor activity (13–17). Due to the increased cytotoxicity of bleomycin and cisplatin at the site of pulse delivery, low doses of chemotherapeutic agents are required, and a good local effect is achieved with no or minimal systemic toxicity. An antivascular effect of the treatment additionally occurs during the treatment; after the pulse application, the so-called “vascular lock” effect is observed, resulting in reduced blood flow and subsequent retention of the chemotherapeutics inside the tumor. Subsequently, the direct destruction of the endothelial cells of small vessels occurs (vascular disrupting effect), further accelerating tumor cell death (18, 19). ECT has also been shown to increase the efficacy of doxorubicin and mitoxantrone (20).

ECT triggers apoptotic and necrotic tumor cell death, and necrosis is expected in the treated area several days after the treatment. The dying tumor cells release extracellular tumor antigens and damage-associated molecular patterns (DAMPs) that trigger cytotoxic immune response against surviving neoplastic cells. This phenomenon is called immunogenic cell death and requires a combination of three DAMPs: calreticulin, which is exposed on the cell, and adenosine triphosphate (ATP) and high mobility group box 1 protein (HMGB1), which are released from dying tumor cells (21).

ECT has been established in more than 140 human clinical centers across Europe and is part of several national guidelines for the treatment of skin tumors in humans, such as the guidelines from the National Institute for Health and Care Excellence (NICE) (9). Recently, the use of ECT in oncology has been successfully expanded to the treatment of deep-seated tumors, such as primary and metastatic liver tumors, colorectal tumors, pancreatic tumors and tumors of the prostate, esophagus, bone, and spinal cord (22–30).

In veterinary medicine, ECT is used for the treatment of cutaneous, subcutaneous and oral tumors in dogs (31–35) and (still mostly) cutaneous tumors in cats (36) and horses (37, 38). In mast cell tumors (MCT) in dogs, up to a 70% complete response rate can be achieved with no major local or systemic side effects (31). In cats with nasal planum squamous cell carcinoma (SCC), the rate of complete responses can be up to 80% (36). The antitumor efficacy is inversely proportional to the tumor size; treating tumors smaller than 2 cm^3^ results in a better complete response rate than treating larger tumors (12, 39). In a study comparing ECT and surgery for treating MCT in dogs, treatment with ECT resulted in a 70% complete response rate, while the rate was 50% for surgical treatment. Thus, ECT offers an alternative treatment to surgery, especially in cases of smaller MCTs and those that are non-resectable because of the location (31). In cases of larger tumors, a combination of surgical removal and intraoperative ECT can improve the treatment outcome (40). ECT is especially suitable for the treatment of solid tumors in cases where animal owners decline surgical treatment, or when tumors are located in areas where complete removal would be impossible (e.g., tumors near large vessels, tumors of the head and limbs, perianal tumors) (12). In veterinary medicine, the first reports of the use of ECT for the treatment of deep-seated tumors were recently described for the treatment of nasal tumors and colorectal carcinomas in dogs and thymoma in a cat (20, 41–43).

For effective treatment, the electric field should be distributed over the entire tumor and its safety margins while avoiding healthy tissues, which may be especially challenging in the oral cavity due to limited access as well as tissues with different conductivities (i.e., soft tissues, teeth, bone), where treatment pre-planning with numerical modeling providing optimal electrical parameters and electrode positions may increase the chances of treatment success (44–46).

### Gene Electrotransfer

Gene electrotransfer (GET) is a method in which plasmid DNA encoding a therapeutic gene is transported into cells by reversible electroporation. In this way, increased production of the desired protein and its release into the extracellular matrix or bloodstream are achieved. The tissues that are most commonly subjected to GET are the skin and muscle. GET can be combined with ECT due to their synergistic action on neoplastic cells; intratumoral ECT directly destroys neoplastic cells, while GET into surrounding tissues transfects healthy cells and, depending on the therapeutic gene, may enhance the antitumor immune response (7, 47).

One of the most widely investigated GET methods in oncology is GET of a plasmid encoding interleukin-12 (IL-12); the safety and efficacy of this treatment has been demonstrated in several pre-clinical studies and translational studies in dogs (48–50). IL-12 has different antitumor effects with direct activation of acquired and innate immunity. It promotes the activation of T cells, enhances T cell survival and the effector functions of T cells and natural killer cells, and promotes interferon gamma (IFN-γ) secretion. IFN-γ acts directly on tumor cells by increasing recognition of major histocompatibility complex 1 (MHC 1), activates M1 macrophages, and enables alteration of the extracellular matrix, resulting in reduced angiogenesis and tumor invasion (50–53). These different actions slow down tumor growth and, finally, destroy the tumor. Additionally, long-term antitumor immunity can be established; in pre-clinical studies in mice with squamous cell carcinoma, the treated animals were resistant to tumor regrowth for 11 months, even when the same tumor cells were administered monthly to the subcutaneous tissues (51). Studies in a murine sarcoma tumor model have shown similar results (54). In addition, in human malignant melanoma and canine squamous cell carcinoma, a systemic “abscopal” effect on untreated tumor and metastases has been observed (55–57).

Following GET of a plasmid encoding human IL-12 (hIL-12), Pavlin et al. (58) evaluated the histological changes in mast cell tumors in dogs. Following intratumoral administration of the plasmid, there was a decrease in the number and degranulation of the tumor cells and extensive infiltration of tumor tissue with lymphocytes and plasma cells (58). Their observations confirmed the findings of previous studies of melanoma in mice, which showed infiltration of helper T cells (CD4+ lymphocytes) and cytotoxic T lymphocytes (CD8+ lymphocytes) after the treatment (59, 60). A similar phenomenon was observed in horses with melanoma treated with intratumoral administration of a plasmid encoding hIL-12 without electrical pulse delivery; in these cases, the infiltration consisted primarily of peritumoral CD4+ lymphocytes (61). Intratumoral infiltration with CD4+ and CD8+ lymphocytes has also been detected in patients with metastatic melanoma treated with GET of a plasmid encoding IL-12 (55).

GET of IL-12 as a monotherapy has demonstrated local and systemic antitumor activity (56, 58, 62), but the success of the treatment can be enhanced if different treatment approaches are combined [e.g., addition of radiotherapy (63) or ECT (7)]. Serša et al. (7) proposed a model using ECT as an *in situ* vaccination, with peritumoral GET of IL-12 to boost the triggered immune response against tumor antigens released from the dying tumor cells due to the action of ECT (7).

The combination of ECT and GET of IL-12 has already been used in the treatment of tumors of different histology in dogs (49, 56, 64, 65). In canine mast cell tumors, the addition of peritumoral GET encoding hIL-12 has been shown to improve the rate of complete response to 72%, compared to 62% for ECT alone (31, 49). Lampreht et al. (66) subsequently developed a plasmid encoding canine IL-12 (cIL-12) with similar or even higher expression capacity than the plasmid encoding hIL-12. This plasmid encoding cIL-12 was subsequently used in the treatment of dogs with oral malignant melanoma (OMM) (65).

Another transgene that was evaluated together with GET was a plasmid encoding chondroitin-sulfate proteoglycan 4 (CSPG4). CSPG4 is a transmembrane glycoprotein that is overexpressed on malignant cells of different tumors and involved in promoting oncogenic transformations, enabling proliferation, motility and metastatic spread of malignant cells via various modes of action. CSPG4 has been recognized as a marker for aggressive, therapy-resistant cancers and simultaneously serves as a target for tumor-selective oncolytic agents [reviewed by Jordaan et al. (67)] and anti-tumor vaccines [reviewed by Rolih et al. (68)]. As CSPG4 displays low expression levels on healthy adult (human and canine) cells and is expressed on the cell surface (Class 1 oncoantigen), it is an ideal target for effective anti-cancer immunotherapy in dogs and humans. A canine model appears to be very useful in translational studies, as CSPG4 expression was found in canine malignant melanoma and osteosarcoma (OSA) (68, 69) and has been related to significantly shorter survival in dogs with appendicular OSA (69). GET of human CSPG4 (hCSPG4) has also been shown to be safe and effective in the treatment of dogs with spontaneous OMM (70, 71); the human sequence of CSPG4 was intentionally used in these studies due to its high homology and similarity to canine CSPG4 and demonstrated ability to induce a specific humoral response against the human and canine protein, which is related to the successful outcome of the treatment (68). CSPG4 immune targeting also appears to be a promising treatment modality for appendicular OSA in dogs, as documented in an *in vitro* model (69).

## Electroporation-Based Treatments for Oral and Maxillofacial Tumors in Small Animals

Electroporation-based treatments (i.e., ECT, GET, and their combinations) have been shown to be safe and effective in veterinary oncology; however, they are not yet widely accepted as standard treatments, as observed to an even greater extent in veterinary oral and maxillofacial oncology. Currently, ECT and/or GET in veterinary oral oncology are recommended mostly for solid tumors for which clients decline surgery and/or radiotherapy (12).

The aims of this paper are to review the currently available data on the use of electroporation-based treatments in veterinary oral and maxillofacial oncology in dogs and cats. Although electroporation-based treatments, especially ECT, are beneficial for the treatment of nasal planum squamous cell carcinoma (SCC) and cutaneous SCC of the head/face and ears in cats (36, 72, 73) and are even more effective for MCT of the skin on the face and ears, lips and conjunctiva in dogs (40), we do not focus on these tumors. We also do not include tumors of the nasal cavity and frontal sinuses in this review.

### Electrochemotherapy for Oral and Maxillofacial Tumors in Small Animals

As early as 2003, Spugnini and Porrello (74) described the principle of potentiation of bleomycin chemotherapy with the application of electroporation in the treatment of different neoplasms without evidence of bone invasion and metastatic disease in different species. Among these patients treated with ECT were also two dogs (one with acanthomatous ameloblastoma (AA) previously addressed with two surgeries and one intralesional chemotherapy, and one with OMM previously addressed with surgery) and three cats (one with oral SCC, one with head fibrosarcoma (FSA) previously addressed with one surgery, and one with an oral anaplastic sarcoma subjected to two previous surgeries). The treatment resulted in a complete response (CR) in the dog with AA (at least 150 days duration) and an initial CR in the cat with anaplastic sarcoma (90 days duration). After an additional ECT treatment upon recurrence of the tumor in this cat, a partial response (PR) lasting 55 days was observed. In the other two cats, a PR of 120 days (SCC) and 14 days (FSA) was reported. The dog with OMM had stable disease (SD) for 40 days. Treatments were generally well-tolerated, although the cat with FSA developed edema at the treated site, and the other two cats developed mild to moderate necrosis at the treated site.

Later, ECT with bleomycin was used by Spugnini et al. (75) in the treatment of 10 dogs with spontaneous OMM without notable metastasis (however, in one dog, metastatic lymph nodes were removed before ECT during surgery) either as a monotherapy (*n* = 4) or after previous surgery resulting in local tumor recurrence (*n* = 6). One week after completion of the treatment (i.e., after four ECT sessions repeated every week), a CR was obtained in seven dogs, and the rest had either SD or a PR. The median survival time (MST) of the dogs was 6 months (mean survival of 16 months). All dogs with either SD or PR eventually developed progressive disease, but four dogs with an initial CR remained in remission for 16–36 months. Moreover, no major local or systemic side effects were noted, except local vitiligo-like discoloration in three dogs, which could potentially indicate recruitment of the immune system by the therapy.

In 2017, Kulbacka et al. (45) described the treatment of one dog with stage IV OMM using a combination of cytoreductive CO_2_ laser surgery and ECT with bleomycin administered intravenously and intratumorally, resulting in the animal's immediate return to function and reduction of the remaining tumor mass within 10 days. However, 14 days after the treatment, enlarged metastatic mandibular lymph nodes caused the dog difficulty eating. At this point, the metastatic lymph nodes as well as the oral tumor were treated with ECT and calcium ions to elicit an additional immune system response. Severe lymphadenitis occurred 5 days post-treatment, but 30 days later no metastases were noted on post-treatment CT. The dog was euthanized 2 months after the first treatment due to seizures.

Suzuki et al. (44) published a case report of an OMM in a dog that was treated with ECT with bleomycin as part of a study to optimize the application of electroporation parameters/electrodes by numerical modeling and measuring oral mucosa conductivity during electroporation. Treatment resulted in clinical CR of the tumor that lasted at least 12 months.

In 2019, Spugnini et al. (76) published results for a study in which 30 dogs with incompletely excised non-metastatic sarcomas not involving bone were treated with ECT combined with bleomycin and cisplatin. Three of these dogs had sarcoma [peripheral nerve sheath tumor (PNST) grade II stage 3, hemangiosarcoma (HSA) stage 2, chondrosarcoma (CSA) stage 2] on the head, although the exact location was not clearly reported in the study. The authors used systemic bleomycin to increase the likelihood of drug distribution in the deeper layers of the tumor bed and local cisplatin to increase the efficacy of the treatment in the superficial layers. In two of the dogs (PNST, CSA) there was no evidence of disease at the end of the observation period [50 months (1,505 days) and 17 months (513 days) post-treatment, respectively], while the dog with HSA was disease-free for 12 months before developing recurrence and metastasis leading to death. Again, all the dogs tolerated the treatment well without major local and systemic side effects.

Torrigiani et al. (34) also treated non-metastatic soft tissue sarcomas in dogs with ECT. Of 52 dogs with 54 spontaneous soft tissue sarcomas, five had a (non-oral) tumor on the head without further specification of the exact location. In contrast to Spugnini et al. (76), the authors only used bleomycin intravenously and compared the efficacy of different treatment approaches–ECT as a monotherapy (performed for macroscopic disease) (*n* = 1), intraoperative ECT (performed immediately after marginal removal of the tumor) (*n* = 2) and adjuvant ECT (for incompletely excised tumors) (*n* = 2). Outcome data specific for head tumors are only available as personal communication with the authors (Torrigiani, personal communication) and hence not included here. Generally, overall response rate for ECT as a monotherapy was 75% and all these dogs were dead at the end of the follow-up period (median 1,113 days). Overall tumor recurrence rate was 23% for dogs treated with intraoperative ECT (median follow-up period 422.5 days) and 25% for dogs treated with adjuvant ECT (median follow-up period 596.5 days). As noted in the previously mentioned studies, treatment was well-tolerated with minor toxicity. In addition, the group compared the treatment outcomes when using two different pulse generators with different ECT parameters. They found no differences in outcomes when comparing the groups treated with different pulse generators, but they observed a higher treatment toxicity score in the group treated with a higher amplitude to electric distance ratio (1,200 vs. 1,000 V/cm) (34).

Recently, two studies on the use of ECT in oral tumors were published for larger cohorts of the patients. Simčič et al. (35) employed ECT with intravenous bleomycin to treat 12 dogs with canine oral non-tonsillar SCC without evidence of distant metastasis at the time of treatment. In that study, only two dogs received two ECT treatments, while the rest were treated only once. The treatment resulted in a calculated response rate for ECT alone of 90.9% and an overall recurrence of 27.3%. This response rate was better than that published for dogs treated with piroxicam alone (response rate of 18%) (77) or the combination of piroxicam and carboplatin (response rate of 57%) (78). However, the recurrence rate was higher compared with that achieved by surgery (8.3–18.2%) (79, 80), but not compared with radiotherapy (39.4%) (81). However, the outcome of the treatment was especially favorable with long-term (median follow-up 1,041 days) CR in dogs (*n* = 6) with tumors smaller than 2 cm compared with larger tumors. Generally, post-operative hypofractionated radiotherapy after incomplete excision of oral SCCs is currently considered to provide the best results (MST of 2,051 days) (82). There was only minor local toxicity (i.e., swelling, necrosis) noted in the majority of cases, and none of the dogs showed signs of systemic toxicity (35).

In the most recent large prospective clinical study using ECT with intravenous bleomycin, Tellado et al. (83) included 67 dogs with OMM of different stages. The animals were rechecked at 14, 30, and 60 days, and dogs with either SD or PD were treated again. Animals were followed-up up to 2 years. The overall objective response (OR) rate was 70.1% with 20.9% CR, 49.3% PR, 16.4% SD and 13.4% PD. The outcome of the treatment was largely dependent on the stage of the tumor; dogs with OMM stages I (*n* = 11) and II (*n* = 19) had a significantly better OR rate (93.3%) than dogs with OMM stages III (*n* = 26) and IV (*n* = 11) with an OR rate 51.4%. Additionally, dogs with OMM stages I (median time to progression 11 months) and II (median time to progression of 7 months) had significantly longer times to disease progression than dogs with OMM stage III or IV (both had a median time to progression of 4 months). The absence of bone invasion was identified as a predictive factor for longer times to progression. Fourteen dogs achieved CR, and the median disease-free survival (DFS) time was 12.5 months (3–30 months). Similarly, the stage was related to the overall survival time, with stage I OMM and absence of bone involvement being predictive of long survival and stage IV being predictive of short survival. The MST for dogs with stage I OMM was 16.5 months, for dogs with stage II OMM 9 months, for dogs with stage III OMM 7.5 months and for dogs with stage IV OMM 4.5 months. Interestingly, none of the dogs treated with stage I OMM developed metastasis, while other dogs that had no metastasis at the time of the first visit subsequently developed metastasis in 23.9% of the cases (83).

Details of the treatments are also summarized in Table 1.

**Table 1:** Studies using electrochemotherapy for the treatment of oral tumors in dogs.

	**Additional treatment**	**Number of treatments**	**Cytostatic used (dosage, administration)**	**Electrodes + electrical pulse parameters (number, duration, amplitude to distance ratio, frequency)**	**Generator of electric pulses**	**Tumor type**	**Number of patients**	**Outcome**	**References**
1	1–2 previous surgeries in 2 dogs and two cats Previous intralesional chemotherapy in one dog	1 in two cats 4 in one cat and 1 dog 6 in 1 dog (1 or 2 weeks apart, repeated until CR or PD)	Bleomycin (intratumorally until saturation)	Caliper electrodes; 8 (biphasic) pulses, 50 + 50 μs, 800 V/cm, frequency not defined	Chemopulse (Center of Bioengineering, Sofia, Bulgaria)	Dogs: AA, OMM Cats: SCC, FSA, anaplastic SA	2 dogs and three cats (22 different animals total with different tumors)	CR 150 days in AA SD 40 days in OMM PR 120 days in SCC PR 14 days in FSA CR 90 days in anaplastic SA	(74)
2	Previous surgery in six dogs	4 (1 week apart)	Bleomycin (intra- and peritumorally, dose/tumor unknown)	Modified caliper and needle electrodes; 8 (biphasic) pulses, 50 + 50 μs, 800 V/cm, 1,000 Hz	Chemopulse (Center of Biomedical Engineering, Sofia, Bulgaria)	OMM	10 dogs	1 week after the 4th ECT: CR 70%, PR 10%, SD 20% MST 6 months, mean ST 16 months	(75)
3	Cytoreductive CO_2_ laser surgery	2 (2 weeks apart)	Bleomycin (0.3 mg/kg IV + intratumorally) in the 1st treatment, calcium ions in the 2nd treatment (5 mM, 10 ml intratumorally)	Two-needle array and Petri Pulser electrodes; 8 square wave pulses, 100 μs, 1300 V/cm, 1 Hz	ECM 830 pulse generator, BTX® (Harvard Apparatus, Holliston, MA, USA)	OMM	1 dog	PR at 1 month	(45)
4	NA	1	Bleomycin (15.000 U/m^2^ body surface area IV)	Type II needle electrodes; 8 pulses, 100 μs, 130 kV/m, 1 Hz	ECM 830 pulse generator, BTX® (Harvard Apparatus, Holliston, MA, USA)	OMM	1 dog	CR (follow-up 12 months)	(44)
5	Neoadjuvant surgery	2 (2 weeks apart) starting 10–14 days after surgery	Bleomycin (20 mg/m^2^ IV) + cisplatin 0.5 mg/cm^2^ in the tumor bed	Plate electrodes; 8 (biphasic) pulses, 50+50 μs, 1300 V/cm, frequency unknown	Onkodisruptor	Head (site not specified) PNST, HSA, CSA	Three dogs (30 dogs total included with sarcomas at other locations)	CR in 2 dogs at 1,505 and 513 days, PD in 1 dog at 366 days	(76)
6	Nothing or previous surgery (marginal or resulting in incomplete margins)	1–3 for 54 tumors (with the intervals between dependent on tumor recurrence)*	Bleomycin (15.000 U/m^2^ body surface area IV)	Type II needle electrodes; 8 (monophasic) pulses, 100 μs, 1,200 V/cm* or 1,000 V/cm*, 5,000 Hz*, 1 Hz*	Cytopulse PA4000 or CytopulseOncovet (Cyto Pulse Sciences, Inc., Holliston)	Head soft tissue sarcoma (non-oral, but site not specified)	Five dogs (52 dogs total included with 54 soft tissue sarcomas at other locations)	Overall response rate for ECT alone 75% Overall RR for ECT alone NA, for intraoperative ECT 23% and for adjuvant ECT 25%* (median follow-up 498 days for all dogs*)	(34) *Data specific to head tumors are available as personal communication Torrigiani, personal communication
7	Surgery before ECT in one case Carboplatin chemotherapy after second ECT in 1 case	1 2 in 2 dogs (1 month apart)	Bleomycin (15.000 U/m^2^ body surface area IV)	type II needle electrodes; 8 (monophasic) pulses, 100 μs, 1,000 V/cm and 1 Hz (PA4000) or 1,200 V/cm and 5 kHz (Oncovet)	Cytopulse PA4000 or CytopulseOncovet (Cyto Pulse Sciences, Inc., Holliston)	SCC	12 dogs	Calculated response rate for ECT alone 90.9%, overall RR 27.3%, DFI and MST for dogs with recurrence 50 days (range 9–83) and 115 days (range 99–1891) Dogs treated with ECT alone with tumors <2 cm obtained CR and showed no recurrence (median follow-up 1041 days)	(35)
8	NA	1 in 41 dogs 2 in 20 dogs 3 in five dogs 4 in 1 dog (with the intervals between dependent on tumor recurrence; usually between 1 and 2 months)	Bleomycin (15.000 U/m^2^ body surface area IV)	6-needle electrodes and Single Needle Electrode®(for nasal duct invasion); 8 (6-needle electrodes) or 32 (single needle electrodes) pulses, 100 μs, 1,000 V/cm, 10 Hz	ECM 830 pulse generator, BTX® (Harvard Apparatus, Holliston, MA, USA)	OMM, stages I–IV	67 dogs	Stage I: CR 72.7%, PR 27.3%; MST 16.5 months Stage II: CR 21.1%, PR 68.4%, PD 10.5%; MST 9 months Stage III: CR 7.7%, PR 50%, SD 26.9%, PD 15.4%; MST 7.5 months Stage IV: PR 36.4%, SD 36.4%, PD 27.3%; MST 4.5 months	(83)

*NA, not applicable; ECT, electrochemotherapy; OMM, oral malignant melanoma; AA, acanthomatous ameloblastoma; SCC, squamous cell carcinoma; SA, sarcoma; FSA, fibrosarcoma; PNST, peripheral nerve sheath tumor; HSA, hemangiosarcoma; CSA, chondrosarcoma; CR, complete response; PR, partial response; SD, stable disease; PD, progressive disease; RR, recurrence rate; MST, median survival time; ST, survival time; DFI, disease-free interval*.

### Gene Electrotransfer for Oral and Maxillofacial Tumors in Small Animals

After successful implementation of ECT and the combination of ECT and GET as described later in veterinary oncology, GET as a mono-gene therapy was also introduced. The first case in veterinary oral oncology was reported by (84), who, as part of the optimization protocol, also treated one dog with oral amelanotic melanoma with GET of a plasmid encoding hIL-12. The treatment consisted of five cycles; in each cycle, the dog received 1–3 GET therapies as detailed in Table 2. The response to each cycle varied, from PR to PD, but finally the local disease was stable after 147 days (further follow-up data are not provided). Moreover, the dog developed metastatic disease (lungs) after the second cycle, but these non-treated metastases stabilized or even regressed with further treatment cycles, although new metastatic lesions developed in the lungs (56).

**Table 2:** Studies using gene electrotransfer for the treatment of oral tumors in dogs.

	**Additional treatment**	**Number of treatments**	**Plasmid DNA (dosage, administration)**	**Electrodes + electrical pulse parameters (number, duration, amplitude to distance ratio, frequency)**	**Generator of electric pulses**	**Tumor type**	**Number of patients**	**Outcome**	**References**
1	NA	5 cycles of 1–3 treatments with at least 6 days between the treatments	hIL-12 (300–600 ug/treatment, intratumorally)	Needle electrode; 2 pulses, 20 ms, 350 V/cm, 10 Hz	ECM 830 pulse generator, BTX®	Amelanotic melanoma (metastatic)	1 dog (4 dogs total included with tumors at other locations)	SD after 147 days; metastases less opaque, smaller, and difficult to identify	(84)
2	Curative-intent surgery 3–4 weeks before 3 dogs later received additional surgery	2 in 2 weeks, then monthly	hCSPG4 (500 ug, IM)	Electrodes unknown; 9 pulses (1 high voltage, 450 V, 50 ms, 3 HZ; 1 s pause; 8 low-voltage 110 V, 20 ms, pause 300 ms)	Cliniporator™ (Igea, Carpi, Italy)	OMM, stage II and III, CSPG4-positive	14 dogs	6-month survival rate 100%, 12-month survival rate 64.3% DFI 477 days MST 653 days Local recurrence 21.4% Lung metastases 35.7%	(71)
3	NA	2–3 treatments (1 day−1 week interval)	hIL-12 (1 mg/treatment, intratumorally)	Needle electrodes; 2 pulses, 0.05 ms, 750 V/cm, 5 kHz 8 pulses, 10 ms, 183 V/cm, 50 Hz	Agile Pulse generator, BTX®	FSA, OSA, SCC (metastatic)	4 dogs (9 dogs total included with tumors at other locations)	Softening of the tumor, but no effect on tumor growth (observation period up to 270 days)	(85)
4	Curative-intent surgery 3–4 weeks before 8 dogs later received additional surgery or radiotherapy	2 in 2 weeks, then monthly; dogs surviving >2 years re-vaccinated every 6 months	hCSPG4 (500 ug, IM)	9 pulses (1 high voltage, 450 V, 50 ms, 3 HZ; 1 s pause; 8 low-voltage 110 V, 20 ms, pause 300 ms)	Cliniporator™ (Igea, Carpi, Italy)	OMM, stage II and III, CSPG4-positive	23 dogs	24-month DFI 17.4%, 24-month survival rate 30.4%, local recurrence 34.8%, lung metastasis 39%	(70)

*NA, not applicable; hCSPG4, human chondroitin sulfate proteoglycan 4; hIL-12, human interleukin-12; OMM, oral malignant melanoma; SCC, squamous cell carcinoma; FSA, fibrosarcoma; OSA, osteosarcoma; SD, stable disease; MST, median survival time; DFI, disease-free interval*.

In the same year, Riccardo et al. (71) introduced GET of a plasmid encoding hCSPG4 as monotherapy to the clinical setting and tested the treatment in 14 dogs with surgically resected (of these three incompletely excised) CSPG4-positive stage II and III OMM. The authors compared this group with the group of 19 dogs with surgically resected stage II and III OMM, of which 13 had CSPG4-positive (4 incomplete excision) and 6 CSPG4-negative (two incomplete excision) OMM. Dogs receiving GET of hCSPG4 had a better survival rate (6- and 12-month survival rates were 100 and 64.3%) than dogs with only surgically resected CSPG4-positive OMM (6- and 12-month survival rates were 69.2 and 15.3%) and then dogs with only surgically resected CSPG4-negative OMM (6- and 12-month survival rates were 83.3 and 33.3%). MST and disease-free interval (DFI) were significantly longer in dogs receiving GET of hCSPG4 (MST 653 days, DFI 477 days) than in dogs with only surgically resected CSPG4-positive OMM (MST 220 days, DFI 180 days), but not longer than in dogs with only surgically resected CSPG4-negative OMM (MST 338 days, DFI 250 days). Apart from transient erythema at the GET site, no other local or systemic side effects were noted. There were no differences in outcome related to the completeness of surgical excision. The treatment resulted in barely detectable circulating T cells reactive to cCSPG4, but there was a marked specific antibody response to hCSPG4 and cCSPG4 in the serum of all dogs, mostly after the second but in all after the third GET treatment. Post-GET sera of most dogs were capable of inhibiting melanoma cell proliferation *in vitro*, although the titer did not correlate with the clinical outcome (71).

In a study by Cicchelero et al. (85), four dogs with (among others) different oral/maxillofacial tumors [FSA (*n* = 2), OSA (*n* = 1), SCC (*n* = 1)], with regional metastatic disease confirmed in the SCC case, were included. Dogs were treated with GET of a plasmid encoding hIL-12. As initial daily treatments resulted in (likely) treatment-related morbidity (immune-mediated anemia) and mortality (fatal thrombocytopenia), further treatments were performed weekly and repeated one or two times. Apart from initial transitory leukopenia, anemia and monocytosis, no other clinically important deviations in hematology and biochemistry were reported. Minor to moderate fatigue, fever, weight loss, anorexia and tumor swelling and pain were also reported in the treated dogs. Although as monotherapy, GET of a plasmid encoding hIL-12 did not result in a clinically relevant suppression of tumor growth, in one dog a significantly increased quality of life was noted. Moreover, the treatment resulted in a local and systemic immune stimulation, decreased blood flow within the tumors and changes suggestive of an anti-angiogenic effect of the treatment (85). The lack of tumor response was probably due to the large size of the treated tumors (one FSA and OSA described as extensive skull invasion).

In the most recent larger cohort study, Piras et al. (70) continued with GET of a plasmid encoding hCSPG4 testing and compared a group of dogs (*n* = 19) with stage II and III CSPG4-positive OMM treated with curative-intent surgery with a group of dogs (*n* = 23) receiving GET of a plasmid encoding hCSPG4 3–4 weeks after the surgery. In each group, there were four dogs in which surgery resulted in incomplete tumor removal. Dogs receiving GET of hCSPG4 had DFI and a better survival rate (statistically significantly for dogs <20 kg) compared with dogs treated with surgery alone (for the GET group, 24-month DFI rate was 17.4% and 24-month survival rate was 30.4%, while for the surgery-only group, 24-month DFI rate was 5.3% and 24-month survival rate was 5.3%). Adjuvant GET of a plasmid encoding hCSPG4 also resulted in lower local recurrence (34.5% for the GET group vs. 42.0% for the surgery only group) and a lower metastatic percentage (39.0% for the GET group vs. 79.0% for the surgery only group). Treatment with GET also resulted in the presence of specific anti-hCSPG4 antibodies in sera in all dogs after the fourth treatment, which was more pronounced in dogs <20 kg (70).

Details of the treatments are also summarized in Table 2.

### Combination of ECT and GET for Oral and Maxillofacial Tumors in Small Animals

The protocol for using a combination of ECT and GET in veterinary oncology described by Cutrera et al. (86) includes a case of a dog with a poorly determined “recurrent papillary tumor with adjacent metastatic bone tumor” on the rostral maxilla, which was treated with a combination of ECT/GET with a plasmid encoding IL-12 (source not mentioned) and bleomycin, both applied intratumorally. Treatment resulted in regression of the visible tumor within 2 weeks after the treatment and complete resolution of the bony lesion at 23 weeks after the treatment (86).

The combination of ECT/GET was also tested on oral SCC (*n* = 2) and one each AA, OMM and oral FSA by Reed et al. (87), who used intratumoral bleomycin combined with feline IL-12 (fIL-12), which is 91% homologous to cIL-12. The authors adjusted the doses of the fIL-12 and bleomycin to the size of the tumor and administered 0.5 IU of bleomycin and 150 μg fIL-12 for each cm^2^ of tumor at the maximum cross-sectional area. There was a good clinical response to the treatment for all tumors−3 dogs had a CR (defined for the study as the disappearance of all measurable tumor at 21 days), and all three dogs were disease-free for at least 9 months (27 months, 56 months). In one dog with SCC, visible tumor disappeared after the second treatment, and the majority of the bone lysis also disappeared after 6 months, with the dog surviving almost 5 years disease-free. According to clinical photos, this is the same dog as described in a protocol by Cutrera et al. (86). Two dogs (OMM, FSA) had a partial response (defined for the study as a >50% reduction in measurable tumor at 21 days) but were soon euthanized due to progressive disease and other medical problems (OMM case) or rapidly recurring local tumor (FSA) without completing the treatment. Apart from transitory leukocytosis, an increase in alkaline phosphatase and diarrhea (the last likely unrelated to the treatment) in the dog with metastatic OMM, and a day of lethargy and decreased appetite in another dog, no major side effects of the treatment were noted (87).

A later report on combined ECT/GET treatment by Cutrera et al. (64) included nine dogs with head and neck AA (*n* = 2), plasmacytoma (PC) (*n* = 1), SCC (*n* = 4), and sarcoma (*n* = 2) (including a subcutaneous sarcoma of the orbital area) with distant metastasis confirmed in the PC case. Four of the dogs were previously treated with surgery or radiotherapy. Intratumoral GET of cIL-12 with or without ECT with either bleomycin or gemcitabine injected intratumorally was performed; these treatments were further repeated based on the tumor response. Bleomycin was the chemotherapeutic agent of choice and was replaced with gemcitabine if the clinical outcome was not favorable. The treatment was divided into cycles composed of one or two treatment sessions 7–28 days apart. The treatments were repeated up to 22 times (12 cycles). The authors concluded that their ECT/GET approach was safe, well-tolerated and similarly effective in reducing the SCC, AA and PC lesion volume within the first 3 weeks after the first treatment, with ECT/GET with bleomycin showing a more rapid effect, especially in SCC cases. However, the treatment was ineffective for sarcomas. Additionally, if only GET was performed, it only temporarily (first 2 weeks with a return to the initial size by the third week) halted SCC growth, but GET alone was more effective for sarcomas than ECT/GET. Interestingly, tumor size was not predictive of the response in cases, that responded to the treatment.

The most recent study on a combination of ECT and GET was published by our group. Nine dogs with histologically confirmed spontaneous OMM stages I to III were treated with cytoreductive surgery (intracapsular excision of the tumors) immediately followed by ECT (intravenous bleomycin) in combination with GET of a plasmid encoding cIL-12 given peritumorally. Treatment was repeated up to five times based on the response to previous treatment(s). The protocol was shown to be safe with no major local or systemic side effects apart from (expected) tumor necrosis and, in some patients, transient systemic leukocytosis. At the end of observation period, all but one animal developed PD with an MST of 6 months, regardless of the tumor stage. We concluded that using a combination of ECT/GET for the treatment of canine OMM is minimally invasive and cost-effective, with a survival comparable to that achieved by radical surgery or radiotherapy, especially for stage II and III tumors (88). Additionally, reduced circulating regulatory T cell numbers were indicative of the systemic antitumor immune response at the end of treatment (65).

Details of the treatments are also summarized in Table 3.

**Table 3:** Studies using the combination of electrochemotherapy and gene electrotransfer for the treatment of oral tumors in dogs.

	**Additional treatment**	**Number of treatments**	**Cytostatic used (dosage, administration)**	**Plasmid DNA (dosage, administration)**	**Electrodes + electrical pulse parameters (number, duration, amplitude to distance ratio, frequency)**	**Generator of electric pulses**	**Tumor type**	**Number of patients**	**Outcome**	**References**
1	NA	1 (unclear from the text)	Bleomycin (0.5 U/cm^2^, intratumorally)	IL-12 (150 ug, intratumorally)	Caliper electrode; 2 pulses, 25 ms, 450 V/cm	BTS EC830 pulse generator	“Recurrent papillary tumor with adjacent metastatic bone tumor”	1 dog	CR 23 weeks after the treatment	(86) [see also (87)]
2	Previous surgery in SCC cases	1–3 treatments (at least 10 days interval)	Bleomycin (0.5–2 IU/treatment depending on the tumor size, intratumorally)	fIL-12 (150 ug−400 ug depending on the tumor size, intratumorally)	Hexagonal electrodes (in one case simple caliper electrodes); 2 pulses 20 ms, 400 V/cm, 10 Hz	ECM 830 pulse generator, BTX®	AA (T2N0M0), 2x SCC (T2bN0M0), OMM (T3bN2bM1), FSA (T3N0M0)	5 dogs (6 dogs total included with tumors at other locations)	CR SCC, CAA (observation period 9–56 months) PR OMM, FSA (exact observation period unknown, but both developed PD soon)	(87)
3	Previous surgery or radiotherapy	Multiple treatments with different frequency and combinations	Bleomycin (100 ul) (1 IU)/cm^3^, intratumorally or gemcitabin (0.5–10 mg/cm^3^, intratumorally)	cIL-12 (2 mg/cm tumor diameter, intratumorally)	Needle electrode; 2 pulses, 20 ms, 350 V/cm, 10 Hz	ECM 830 pulse generator, BTX®	AA, PC, SCC, sarcoma	9 dogs (13 dogs total included with tumors at other locations)	27% volume reduction in, SCC and PC (in 3 weeks); 165% volume increase in sarcomas (exact observation period unknown)	(64)
4	Neoadjuvant marginal surgery	1–5 treatments (2–4 weeks interval)	Bleomycin (0.3 mg/kg once IV)	cIL-12 (2 mg per treatment, peritumorally)	Plate or needle electrodes; ECT (8 pulses, 100 μs, 1,300 V/cm, 5 kHz) Multielectrode array (MEA) electrode; GET (24 pulses, 150 ms, 60 V, 4 Hz)	Cliniporator™	OMM, stage I, II and III	9 dogs	One month after the treatment: CR 33%, PR 33%, PD 33% End of observation period (2–22 months): CR 11%, PD 89% MST 6 months	(65)

*NA, not applicable; ECT, electrochemotherapy; GET, gene electrotransfer; fIL-12, feline interleukin-12; cIL-12, canine interleukin-12; CR, complete response; PR, partial response; PD, progressive disease; MST, median survival time; OMM, oral malignant melanoma; AA, acanthomatous ameloblastoma; SCC, squamous cell carcinoma; FSA, fibrosarcoma; PC, plasmacytoma; MST, median survival time*.

## Concluding Recommendations on Electroporation-Based Treatments for Oral and Maxillofacial Tumors in Small Animals

There are three major advantages of electroporation-based treatments. First and most important, current treatments offer a safe alternative for the treatment of oral and maxillofacial tumors. Considerable muscle contractions that are consistently encountered during electroporation are expected; hence, ECT/GET treatments must be performed under general anesthesia. Although ECT can be effective as a one-time treatment, several treatments and therefore anesthetic procedures may be needed several weeks apart to increase effectiveness in the case of no response to treatment or only a partial response is achieved (12). Occasional and manageable increases in respiratory and heart rates are also observed during anesthesia. In the post-operative period, transitory mild blood changes may occur, as well as local tumor swelling and necrosis. Generally, ECT/GET treatments are well-tolerated, with most of the dogs in all studies maintaining their normal habits and routines (56, 64, 65, 70, 71, 85, 87). Conversely, Torrigiani et al. (34) reported higher local treatment toxicity if a higher amplitude to electric distance ratio (1,200 vs. 1,000 V/cm) was used with type II needle electrodes. However, the possible post-treatment complications can be considered much less severe than those of surgery for oral tumors (79) or radiotherapy (89, 90). However, it should be noted that data from some other studies point at possible more severe or even fatal complications associated with electroporation-based treatments (85). Local intratumoral application of plasmid encoding IL-12 may cause late focal kidney inflammation without any hematological or biochemical markers of kidney failure in treated animals, as described in mice (91). Although no such reports are available for dogs and cats, monitoring of renal function is recommended in any IL-12 gene-based therapy. In a study including different animals with different types of tumors treated with ECT, acute tumor lysis syndrome and fatal pulmonary thromboembolism were reported in a few cases with large non-oral carcinomas and sarcomas (74).

Further, these treatments, especially ECT, are considered simple and short with no major equipment needed apart from pulse generator, requiring a low dose of chemotherapeutic drugs to produce minimal chemotherapy-related side effects. Therefore, they can be performed on an outpatient basis, further reducing the costs of treatment (12, 20). ECT could therefore be easily offered as an alternative treatment to surgery and radiotherapy, mostly when owners have concerns about the financial burden and/or aesthetic outcome. Conversely, the commercial availability of plasmids for performing GET treatments remains a limitation to the wider application of this procedure.

Finally, when recommending cancer treatment, clinicians need to successfully manage clients' expectations, especially with regard to their animal's well-being. In this respect, electroporation-based treatments offer a treatment option that mostly results in an increased quality of life of the patients, despite observed tumor necrosis (expected) and transient pain of ~2 weeks' duration. There are no occurrences of nausea or gastrointestinal problems and mostly no changes in the normal habits of the animal (35, 40, 56, 64, 65, 85). Moreover, most of the owners (86.4%) of the 44 dogs opting for ECT/GET treatment for their dog reported that they would opt for the same treatment modality again as they assessed the health-related quality of their dogs' life improved 1 month after the treatment (as per RECIST criteria the optimal time to evaluate response); additionally, for those owners, dogs receiving the ECT/GET treatment had an OR (92). While this phenomenon may be true for dogs bearing cutaneous or subcutaneous tumors, the owners of six dogs with oral tumors enrolled in the study reported a worsened health-related quality of life; this was expected as most of these cases were treated with a combination of surgery and ECT/GET and as the response rates of oral tumors to ECT/GET are still lower than cutaneous and subcutaneous tumors (92). However, Tellado et al. (83) recently specified further that quality of life of dogs with OMM treated with ECT improved, but only for dogs with tumor stages I–III, and only if CR or PR was achieved.

In terms of the success of electroporation-based treatments for oral and maxillofacial tumors in small animals, recommendations to clients should be given with some precautions. Namely, data obtained from the studies to date are difficult to interpret due to mostly small sample sizes, inconsistencies in staging of the oral tumors, different treatment protocols and lack of data for cats (Tables 1–3). Another issue that makes comparisons between outcomes of the studies difficult is the discrepancy among the studies in evaluations of the tumor response; some studies, for example, define CR as the disappearance of all evidence of tumor, PR as a decrease in tumor size by at least 50%, SD as a decrease of <50% or an increase of 25% and PD as an increase of the tumor by 25% (74, 75), while others consider PR as a ≥30% reduction in tumor diameter, SD <30% reduction in tumor diameter or <20% increase in tumor diameter and PD ≥20% increase in tumor diameter (34, 65); in further studies, the distinctions were unclear (83). To improve the quality of reporting clinical studies from this field, recommendations for reporting were published in human oncology (93), which could be extrapolated to veterinary use as well.

**Figure 4:**
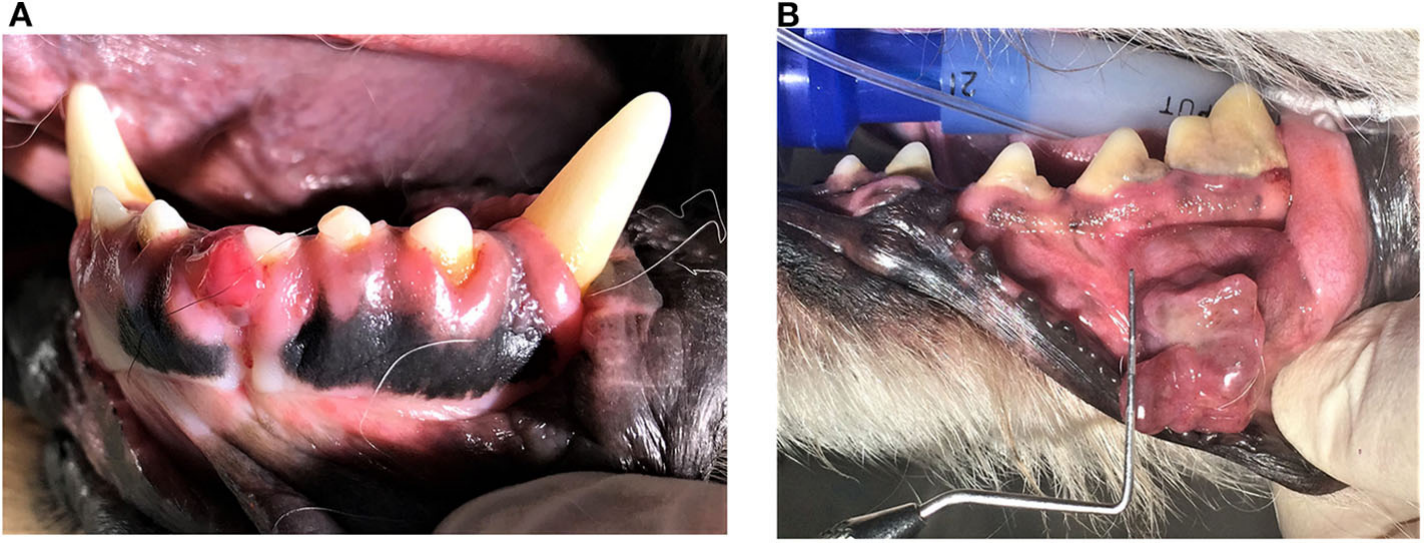
Clinical example of a small squamous cell carcinoma (SCC) affecting the gingiva at the right mandibular first incisor tooth **(A)** and malignant melanoma of the buccal mucosa **(B)** in a dog. Appropriate staging of the disease (biopsy of the lesion, evaluation of the local disease extent and regional lymph nodes and distant organs metastasis employing advanced imaging techniques) is needed before any treatment and prognosis are discussed.

Within these limitations, however, it can be safely concluded that the best local response with ECT is reported for small oral tumors (SCC and OMM, Figure 4), which is likely related to the nature of the treatment (size of the electrodes) as well as the reduced possibility of necrosis, which affects the distribution of the chemotherapeutic (35, 83). Similarly, tumors not involving the bone are easier to treat due to the easier insertion of electrodes (83) and similar tissue conductivity (44). It must be stressed that ECT is a local ablative treatment without a noticeable/expected effect on distant metastasis and should therefore be combined with other treatments in cases of metastatic disease (12). If ECT is combined with GET or GET performed as a monotherapy, a systemic therapeutic effect is expected (and observed in some studies as systemic immune stimulation), but studies to date show no confirmed effect on distant metastasis (85) or a response that is mixed at best (56). Combination with other treatments, such as surgery, radiotherapy, immune checkpoint inhibitors, antiangiogenic therapy or metronomic cyclophosphamide therapy, have also been suggested by several authors (40, 64, 65, 70, 71, 76, 85), but further studies are needed combining different treatment modalities. Additionally, ECT should be used with caution in previously irradiated fields due to the possibility of the animals developing radiation recall (94). However, in human oncology, several studies have evaluated tumor treatment in previously irradiated fields. The major conclusion from these studies is that the antitumor effectiveness of ECT is lower in previously irradiated tumors than naïve tumors, also resulting in more severe necrosis and inflammation. However, no studies have reported major radiation recall; thus, it can be expected that this will not develop in veterinary patients (95, 96).

## Future Trends of ECT/GET in Veterinary Oral and Maxillofacial Oncology

Although ECT, GET and their combination are already used for the treatment of client-owned dogs and cats with cancers of different origins and at different locations (32, 49, 65), several open questions remain.

It has been well-established that ECT can be effective as a one-time treatment, and that if it is not, further ECT treatments can be performed, but there is currently no consensus on when is the best time for retreatment (12, 83). Similarly, electroporation condition optimization for oral tumors is needed (44, 85).

Candidate genes for GET, the location of delivery and the dose of the plasmids encoding candidate genes as well as the dose of chemotherapeutics also require further investigation. While GET with peritumoral delivery of a plasmid encoding IL-12 has already shown promising results in the treatment of different tumor types in dogs (49, 65, 97), the effect of intratumoral application of the plasmid must be established. Similarly, bleomycin pharmacokinetics has to be better understood; in elderly human patients treated with ECT, a lower bleomycin dose was recommended based on pharmacokinetic studies (98), as ECT with a lower bleomycin dose showed comparable antitumor efficacy to using a standard dose (99, 100).

**Figure 5:**
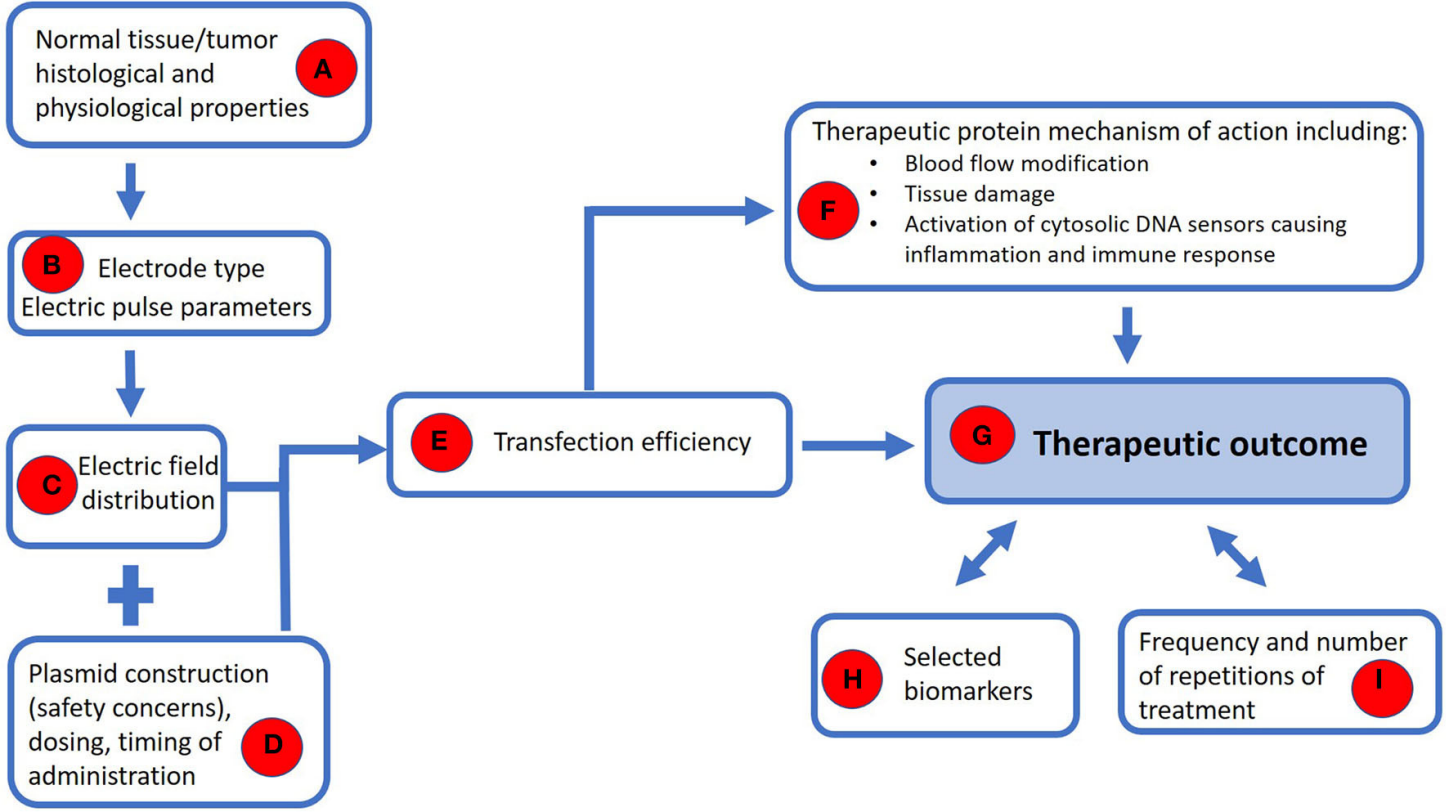
A model for safe and effective use of the GET with further guidance for its wider clinical use. The histological and physiological properties of the tumor and its surrounding normal tissue potentially involved in the neoplastic process (e.g., bone) **(A)** dictate selection of the type of electrode and the parameters of electric pulses **(B)** in order to achieve an appropriate distribution of the electric field **(C)**. A safe plasmid should be used at the appropriate dose and time window prior to the application of electrical pulses **(D)** to ensure sufficient transfection **(E)** that would lead to the production of therapeutic protein **(F)**. The mechanism of action of the therapeutic protein takes place on several levels–through blood flow modification and tissue damage, thereby activating DNA sensors in the cytosol, leading to an inflammatory and immune response **(F)**. To achieve a therapeutic outcome **(G)**, we need to monitor selected biomarkers **(H)**, based on which we could determine the appropriate frequency and number of repetitions of treatment **(I)**.

Finally, the reasons for clinically observed individually different responses to treatment remain to be elucidated with the identification of biomarkers that will enable the better selection of patients that will benefit from the treatment and prediction of response and recurrence (Figure 5). Specific T cell populations in whole blood can be potentially used as biomarkers for early recurrence, showing that additional treatments should be applied (101, 102). High expression of immune checkpoint inhibitors, such as PD-1 and PD-L1, as well as their binding and consecutive immune evasion of the tumor, could also be used as predictive factors for the response to immunotherapy (103, 104). Moreover, the response to immunotherapy of different tumors seems to be specific to the individual, and in particular, the intestinal microbiota is considered an important immune response modulator in human oncologic patients (105). Research has shown that in human melanoma patients, the success of immunotherapy correlates/varies with the composition of the intestinal microbiota (106, 107). This phenomenon could be of a great importance in implementing personalized therapeutic protocols (108). In dogs, a difference in intestinal microbiota composition has been reported between those with colorectal epithelial tumors (109) or lymphoma (110) and healthy individuals. However, there is currently no information on peripheral solid tumors and the role played by the gastrointestinal microbiota in the response to immunotherapy in dogs.

## Author Contributions

All authors listed have made a substantial, direct and intellectual contribution to the work and approved it for publication.

## Conflict of Interest

The authors declare that the research was conducted in the absence of any commercial or financial relationships that could be construed as a potential conflict of interest.
